# Calcium-binding properties, stability, and osteogenic ability of phosphorylated soy peptide-calcium chelate

**DOI:** 10.3389/fnut.2023.1129548

**Published:** 2023-04-21

**Authors:** Xiao Kong, Ziqun Xiao, Yuhang Chen, MengDi Du, Zihui Zhang, Zhenhua Wang, Bo Xu, Yongqiang Cheng, Tianying Yu, Jing Gan

**Affiliations:** ^1^Center for Mitochondria and Healthy Aging, College of Life Science, Yantai University, Yantai, Shandong, China; ^2^Beijing Key Laboratory of Functional Food from Plant Resources, College of Food Science and Nutritional Engineering, China Agricultural University, Beijing, China; ^3^School of Food Science and Technology, Jiangnan University, Wuxi, Jiangsu, China

**Keywords:** phosphorylation, peptide-calcium chelate, characterization, thermal stability, osteogenic differentiation, calcium supplement

## Abstract

**Introduction:**

Bioactive peptides based on foodstuffs are of particular interest as carriers for calcium delivery due to their safety and high activity. The phosphorylated peptide has been shown to enhance calcium absorption and bone formation.

**Method:**

A novel complex of peptide phosphorylation modification derived from soybean protein was introduced, and the mechanism, stability, and osteogenic differentiation bioactivity of the peptide with or without calcium were studied.

**Result:**

The calcium-binding capacity of phosphorylated soy peptide (SPP) reached 50.24 ± 0.20 mg/g. The result of computer stimulation and vibration spectrum showed that SPP could chelate with calcium by the phosphoric acid group, carboxyl oxygen of C-terminal Glu, Asp, and Arg, and phosphoric acid group of Ser on the SPP at a stoichiometric ratio of 1:1, resulting in the formation of the complex of ligand and peptide. Thermal stability showed that chelation enhanced peptide stability compared with SPP alone. Additionally, *in vitro* results showed that SPP-Ca could facilitate osteogenic proliferation and differentiation ability.

**Discussion:**

SPP may function as a promising alternative to current therapeutic agents for bone loss.

## 1. Introduction

Calcium is an essential element that is crucial for the maintenance of bone health. The deficiency of calcium leads to rickets in children and osteoporosis in adults (1, 2). Numerous calcium supplementation on the market emerged, but many questions remain for various calcium salts with stability and absorption. Ion calcium supplements are easily influenced by phytates, oxalate, and non-fermentable fiber of food and may induce the formation of insoluble calcium salt precipitation under intestinal conditions (3). Thus, calcium-binding peptides from foodstuff became a new source of calcium-enriched nutrients with the potential to overcome the limitations of simple calcium intake.

Bioactive peptides normally contain 2 to 20 amino acid residues and are abundant in hydrophobic amino acids (4). These bioactive motifs exhibited properties such as antihypertensive, antimicrobial, and excellent health effects as antioxidants (5–7). It has been reported that bioactive peptides could chelate with calcium ions to form stable complexes with superior absorptivity based on mineral binding functions (8, 9). Nowadays, various calcium-binding peptides have been obtained from food resources, including phosvitin hydrolysates, porcine plasma protein hydrolysates, and wheat germ protein hydrolysates (10–12). Among them, casein phosphor-peptides have been reported to be desired mineral carriers that can strengthen elemental mineral absorption, mostly chelated with calcium through the negatively charged phosphate group (13).

In our preliminary experiments, DEDEQIPSHPPR dodecapeptide from soy yogurt was confirmed to possess 36.64 ± 0.04 mg/g calcium-chelating capacity (14). Previous studies found that the calcium chelation capacity is closely related to their molecular mass, amino acid compositions, sequences, and spatial conformation (15, 16). The carboxyl groups of Asp and Glu, the δ-N in the imidazole ring of His, and the ε-amino nitrogen of Lys are considered to play a critical role in calcium binding (17). Among them, His mainly depends on the accumulation of cyclic side chains, and nitrogen atoms on the imidazole ring can also be used as hydrogen bond donors and acceptors with calcium ions under different conditions to improve calcium-binding ability (18). However, our study showed that the binding ability of His is obviously lower than that of Asp and Glu (19). Additionally, the primary structure of SPP contains serine residues with free hydroxyl groups, which are sites for the modification with the phosphate group, thus contributing to calcium-binding capacity (20). Luo et al. (21) also confirmed that factors such as phosphate residue and amino acid composition on serine and molecular structure affect the calcium absorption activity and mechanism of CPP. Therefore, we tried to phosphorylate soybean peptides to improve calcium-binding capacity.

Taking all of these into account, the main purpose of this study was to prepare phosphorylated soy peptides calcium complex, to explore its calcium-binding mechanism by computational docking and spectroscopic methods, to investigate the thermal stability and the ability to promote osteogenic proliferation and differentiation *in vitro*. The study could provide comprehensive descriptions of developing new food therapy options to prevent calcium deficiency.

## 2. Materials and methods

### 2.1. Materials

Polypeptide SPP isolated from soy yogurt with a purity of 98% was synthesized by Nanjing Peptide Industry Biotechnology co., ltd. Bile salts, trypsin, and pepsin were products of Sigma–Aldrich (St. Louis, MO). All commercial reagents were of analytical grade.

### 2.2. Molecular dynamic simulation

The Desmond program was performed to study high-performance molecular dynamic simulation properties for SPP and calcium. The initial SPP structure was parameterized in Maestro 11.8 with Epik protonation, following conducting energy minimization at pH 7.0. Then, the solvent system was developed using the system builder platform under SPC mode building an explicit solvation cube with a 10Å × 10Å × 10Å margin added 0.75 mol/L Ca^2+^. Meanwhile, the cube was injected with Cl^−^ as a counter ion to equalize the charge on the OPLS_3E force field. The peptide calcium complex system was initially balanced for 100 nanoseconds (ns) using an NVT ensemble. The simulation process is as follows: step 1, Brownian dynamics NVT, T = 10 K, small time steps, restraining on heavy solute atoms, 100 ps; step 2: NVT, T = 10 K, small time steps, controlling of heavy solute atoms, 12 ps; step 3: NPT, T = 10 K, restraining on heavy solute atoms, 12 ps; step 4: NPT, restricting on solute heavy particles, 12 ps; step 5: NPT, no restrictions, 24 ps; step 6: NPT, no restraints, 100 ns. Subsequently, the system stability was determined by analyzing the MD simulation results of 100 ns, including calculating root mean square deviation (RMSD), root mean square equation (RMSF), the radius of gyration (Rg), and the number of hydrogen bonds (H-bonds).

### 2.3. Calcium-binding capacity analysis

After synthesis, 3 mg/mL SPP and 5 mM CaCl_2_ were mixed in 20 mM sodium phosphate buffer solution (pH = 7.8) maintained at 40°C for 30 min through a shaker water bath, followed by dialysis to remove other elements and centrifugation (4,000 g, 4°C, 20 min). We collected supernation to determine calcium content inductively coupled plasma-atomic emission spectrometry (ELAN DRC II, PerkinElmer, Waltham, United States).

### 2.4. Fabrication of peptide–calcium chelate

Peptide–calcium complex preparation was performed following the method of Cai with slight modification (16). In brief, the stock solution of SPP (10 mg/mL) was mixed with CaCl_2_ at a ratio of 2:1. The pH was adjusted to 8.0 using 1 M NaOH dropwise. After that, the mixture of SPP/CaCl_2_ was shaken at 37°C at 140 rpm in a water bath for 20 min, and absolute ethanol (90 mL) was added to remove the free calcium. The final solution was, then, placed in a dialysis bag for 48 h and centrifuged for 10 min at 10,000 g. The collected precipitation was lyophilized in a vacuum desiccator (ALPHA1-2LDplus, Marin Christ, Osterode, Germany), and thus, the peptide–calcium chelate was prepared for subsequent experiments.

### 2.5. Isothermal titration calorimetry (ITC)

Isothermal titration calorimetry was tested for the characterization of the thermodynamic parameters of SPP binding with calcium. Both the peptide and the SPP-Ca chelate of SPP were dissolved in a Tris-HCl buffer solution. The sample was degassed followed by filtration through a 0.22 μm filter. 2.5 mM of DEDEQIPSHPPR was loaded into the ITC cell, and then, the CaCl_2_ solution (50 mM) was loaded into the ITC syringe. During measurements, 20 drops were injected into the sample cell. The titration parameter was configured to inject CaCl_2_ every 5 min with volumes of 2.5 μL. Nano Analyze software (TA Instrument-Waters LLC, New Castle, Delaware) was applied for evaluating the raw data based on data fitting.

### 2.6. Analysis of binding sites of calcium on SPP

#### 2.6.1. Ultraviolet spectroscopy

The ultraviolet absorption spectra of SPP and SPP-Ca were determined utilizing an ultraviolet spectrophotometer (UV-1200, Xiaofen Instrument Co. Ltd., Guangzhou, China). A stock solution of the peptide was prepared (0.02 mg/mL, pH = 8.0), and then 0.2, 0.4, 0.6, and 0.8 mM of CaCl_2_ were constantly shaking at 50°C for 1 h, and the measurement was conducted over the wavelength ranged from 190 to 400 nm.

#### 2.6.2. Fourier transform infrared spectroscopy

Infrared spectra of samples were obtained by an infrared spectrophotometer (IS50, Thermo Nicolet Co., Waltham, MA) within a scope of 4,000 to 400 cm^−1^. Pellets were created by mixing the sample with KBr and pressing them to form disks. Circular Dichroism Spectroscopy.

Phosphorylated soy peptide (SPP) and SPP-Ca chelate were dissolved in distilled water, making a final concentration of 0.5 mg/mL. The CD spectra were searched by a Chirascan-plus CD spectrometer (Applied Photophysics, Surrey, UK). All experiments were taken over from 200 to 260 nm with programmed 0.5 nm intervals at 25°C.

### 2.7. Thermal stability analysis of peptide–calcium chelate *in vivo*

A thermogravimetric/differential scanning calorimeter (STA449C, Netzsch, Germany) was used to investigate the thermal decomposition of SPP-Ca chelate. Approximately 5 mg of lyophilized sample was weighed and heated from 40 to 600°C with a heating rate of 20°C/min and Nitrogen flow rate of 30 mL/min.

### 2.8. Cell proliferation assay

The influence of the SPP-Ca chelate on MC3T3-E1 cells promoting osteoblast proliferation was quantified using an MTT assay proposed by Wang et al. (22). In brief, MC3T3-E1 cells at a density of 1 × 10^4^ cells/well were grown in 96-well tissue culture plates for 24 h. The medium was replaced by SPP at concentrations of 0, 0.7, 7, and 70 μM and SPP-Ca at a concentration of 70 μM and incubation for 72 h. For survival assay, the cells were treated with 10 μL MTT solution (5 mg/mL in PBS), and the plate was kept in the incubator for 3 h. After that, 150 μL DMSO was added into each well followed by shaken 96-well plates for 10 min. Finally, the optical density was determined at 570 nm by a microplate reader (Molecular Devices, San Jose, CA, United States).

### 2.9. ALP activity assay

Alkaline phosphatase (ALP) activity is a recognized osteogenic differentiation marker of MC3T3-E1 cells. Cells were grown in a complete medium at a density of 1 × 10^4^ cells/well and cultured for 48 h. Then, the medium was changed to a differentiation medium. After 4 days of incubation, fresh serum containing various concentrations of SPP (0.7, 7, and 70 μM) and SPP-Ca (70 μM) was added and kept for 24 h at 37°C. Next, the cells were rinsed with PBS buffer, lysed with 70 μL of ice-cold lysis buffer which was supplemented with PMSF per well, and broken down using a cell disruptor (BiLon 92-II, Beijing, China). The ALP activity and protein concentration were tested using an Alkaline Phosphatase Assay Kit at OD405 and a BCA Protein Assay Kit at OD562 nm.

### 2.10. Statistical analysis

Each experiment was carried out in triplicate, and data were presented as the mean ± SD. The results were subjected to one-way analysis of variance (ANOVA) by the program SPSS Statistics 26.0 (SPSS Institute, Chicago, IL, United States), and the significant statistical differences were set at *p* < 0.05.

## 3. Results

### 3.1. Molecular dynamic simulation of SPP-Ca

**Figure 1:**
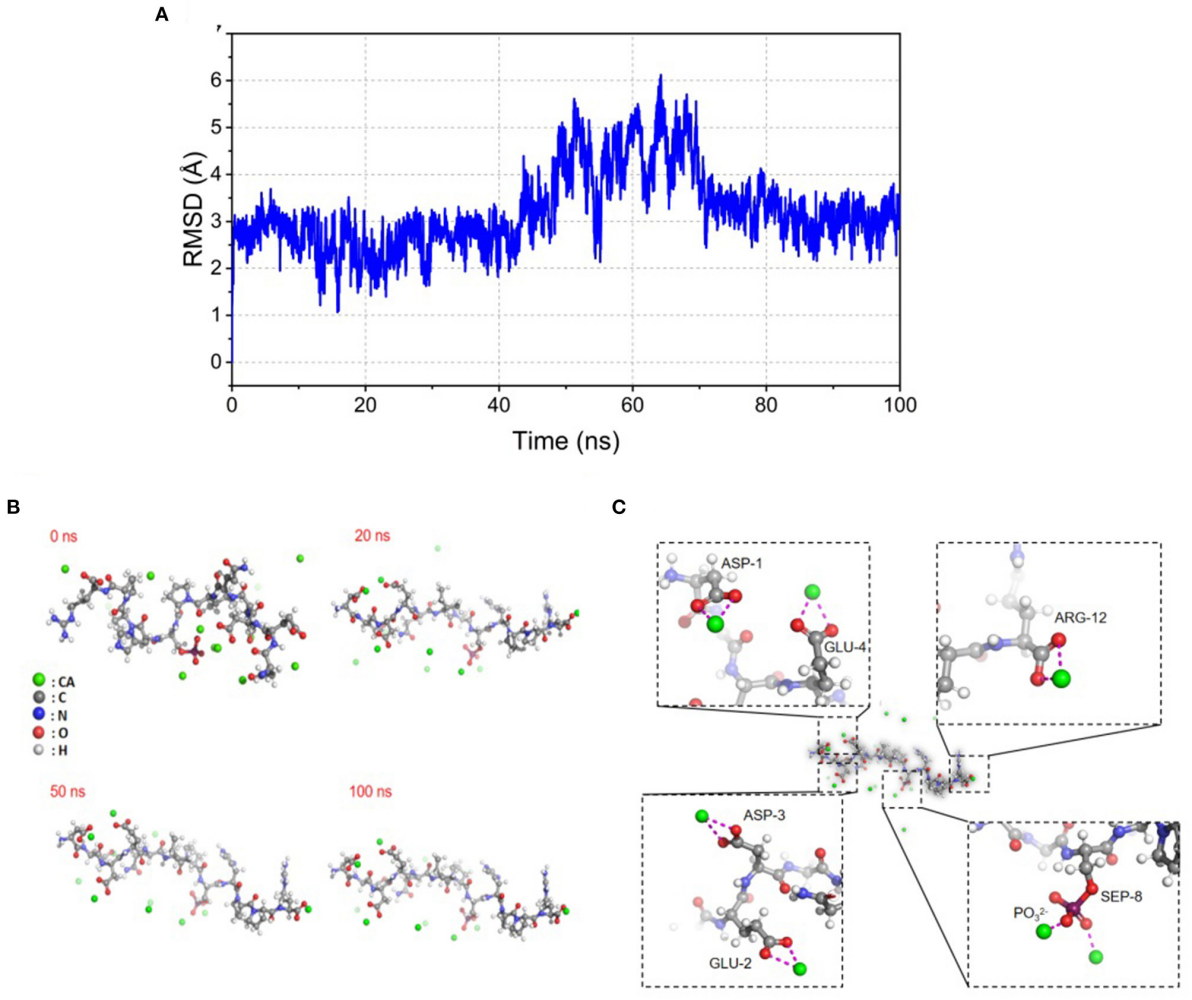
Illustration of molecular dynamic analysis simulation for SPP-Ca. **(A)** RMSD stability analysis, changes of RMSD of SPPs with simulated time during molecular docking; **(B)** snapshots of molecular dynamics simulation of SPP-calcium chelate at (0, 20, 50, and 100 ns), the green ball represents Ca^2+^, the gray ball represents carbon atom on amino acid, the blue ball represents nitrogen atom on amino acid, the red ball represents oxygen atom on amino acid, and the white ball represents hydrogen atom on amino acid; **(C)** molecular dynamic analysis simulation chelation of SPP-Ca complex based on density functional theory calculation in the Materials Studio 6.0 software package at 100 ns.

Molecular docking was used to visualize the binding site and patterns between the small molecule ligand and the receptor (23). In this study, molecular docking technology was carried out to predict bound conformations of binding for Ca^2+^ to SPP at a molecular level. Materials Studio 6.0 software package was performed to calculate the docking results accurately. The structure of peptides was optimized on a model based on the density functional theory. The molecular dynamics simulation snapshot (0, 20, 50, and 100 ns) of SPP-calcium chelate is shown in Figure 1A. The RMSD presented a significant tendency to be stable ranging from 2 to 6 Å with the increasing of snapshot. As expected, SPP has undergone a marked conformational change, with various number of contiguous ion binding sites ranging from 0 to 5. For 0 ns, calcium ions were uniformly distributed in the energy minimization system. Subsequently, the number of binding sites gradually increased and reached a stable value of 5 after 100 ns (Figure 1B). Many studies revealed that the presence of negatively charged acidic amino groups was responsible for the covalent bonding of metal ions (4, 12), which was also supported by Figure 1C. In addition, phosphate groups are also involved in chelation. Thus, the result demonstrated the possibility of SPP binding to calcium ions.

### 3.2. Determination of the binding stoichiometry and binding constant with SPP-calcium complex

#### 3.2.1. Analysis of the calcium-binding activity of the SPP

In our study, SPP was confirmed to possess a calcium-binding capacity of 50.24 ± 0.20 mg/g. Moreover, the molecular weight of SPP was 750.65 Da determined by LC-MS, as shown in the Supplementary Figure 1, supporting prior research that the peptide with low molecular weight demonstrates an enhanced affinity to calcium (24, 25). The high proportion of acidic amino acids in SPP also has been reported to contribute to calcium chelation (26, 27). Therefore, SPP could act as an effective carrier in delivering calcium.

#### 3.2.2. Determination of the binding stoichiometry and binding constant

**Figure 2:**
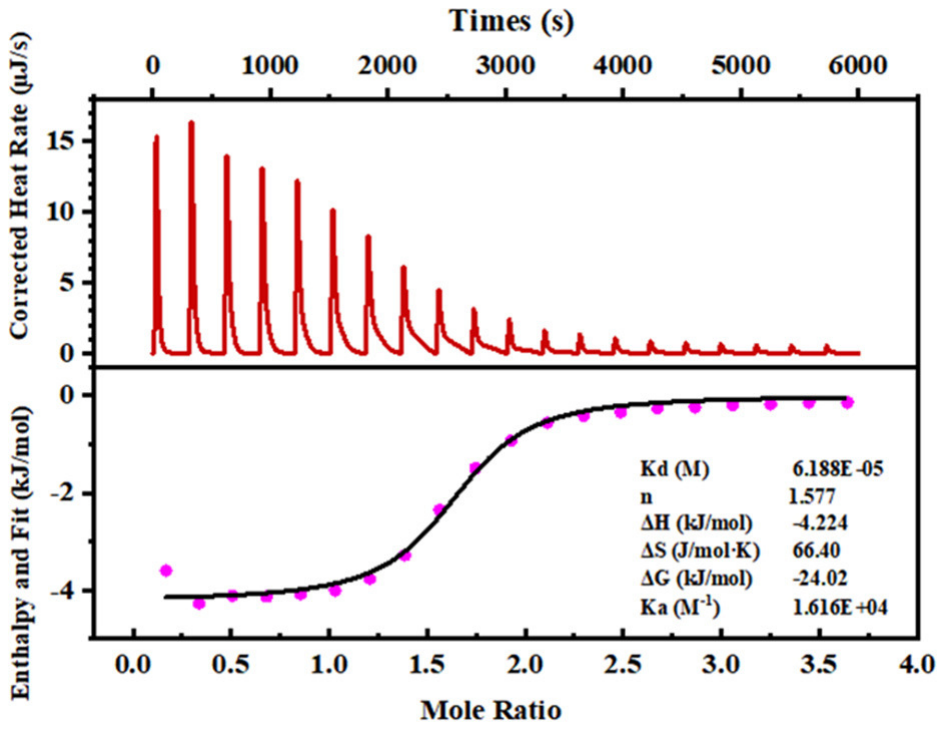
ITC analyses of the SPP reacting with calcium ions. The upper panel exhibits a representative calorimetric titration curve. CaCl_2_ (50 mM) was titrated into 2.5 mM of the peptide solution at 25°C. The lower panel shows the integrated areas corresponding to each titration, plotted as a function of the Ca^2+^/peptide molar ratio. The solid line represents the best curve fit obtained by using an independent binding site model.

Isothermal titration calorimetry (ITC) is a powerful technique for understanding the interaction of ligand-binding peptide (28). The thermodynamic parameters showed that binding to calcium generated negative changes in enthalpy and entropy values (ΔH = −2.224 kJ/mol, ΔG = −24.02 kJ/mol), which demonstrated that the reaction occurs spontaneously and is an exothermic process (Figure 2). In addition, the exothermic reaction yielded by the formation of a coordination bond between Ca^2+^ and phosvitin exerted a negative effect on ΔH. Meanwhile, the high absolute value of ΔS (66.40 J/mol·K) revealed that the association process was electrostatic interaction-driven. The estimated stoichiometric ratio (n) was 1.577, manifesting that the stoichiometric ratio of peptide and calcium was 1:1.

### 3.3. Mechanism of SPP-Ca chelation by spectroscopy

#### 3.3.1. Ultraviolet spectroscopy analysis

**Figure 3:**
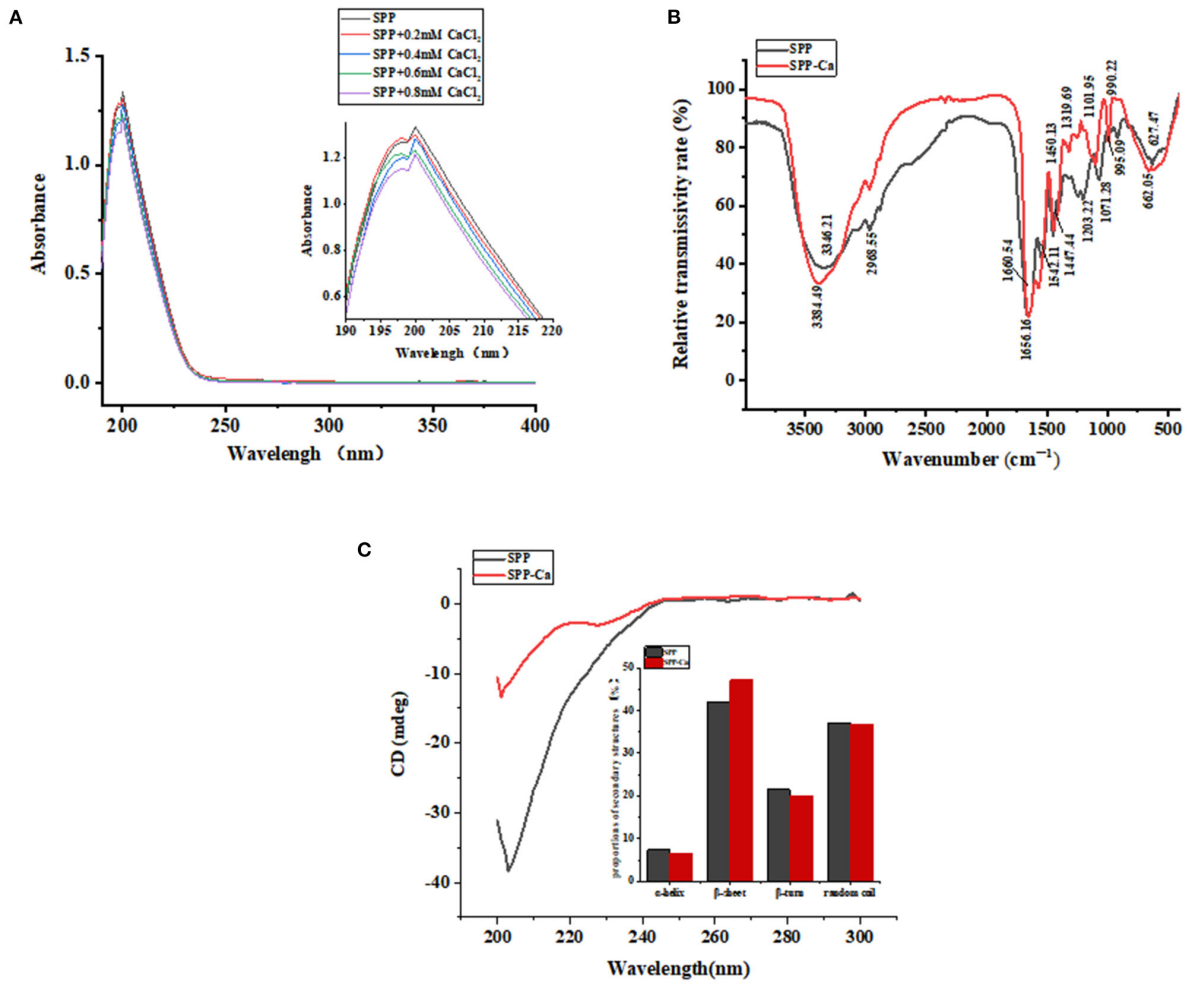
UV spectra **(A)**, FTIR spectra **(B)**, and circular dichroism spectra **(C)** of SPP and SPP-Ca.

Ultraviolet-visible (UV) spectroscopy is recognized as an excellent method to analyze peptide conformational changes induced by the binding of calcium. The peptide–calcium complex formation involving organic ligands and metal ions could produce different UV spectra because of the transfer or disappearance of the original absorbance peaks or the emergence of new absorption peaks (15). Figure 3A shows that SPP has a specific absorption peak at ~200 nm, representing the characteristics of *n* → π^*^ in the amide bond. After cooperating with calcium, it can be observed that the absorption band intensity of the amide bond increased significantly from 1.227 to 1.338 in the magnified UV spectra image, suggesting a hyperchromic effect. The transformation may be due to polarization changes generated by auxochrome groups (–OH, –NH_2_) and chromophore groups (–C = O, –COOH) (29). Hence, it could be concluded that the nitrogen atom of –NH and –NH_2_, the oxygen atom of –C = O and –COOH, and the phosphate group of SPP might be involved in calcium binding.

#### 3.3.2. FTIR analysis

The FTIR analysis could be utilized to observe characteristic absorption peak variations in position, peak number and intensity of amides and carboxylates, and further to reflect the interaction between ligand and metal ion (30). With this in mind, we attempted to explore changes induced by chelation in SPP by FTIR analysis.

As presented in Figure 3B, the dominant spectral peaks are contributed by the amide I (1,700–1,600 cm^−1^) ascribed to C = O bonds stretching and amide II groups (1,600–1,500 cm^−1^) and assigned to the folding of N–H bonds and stretching of C–N bonds, respectively (31, 32). In the spectra of SPP-Ca, the band at 1,660.64 and 1,542.11 cm^−1^ shifted to 1,656.16 and 1,572.18 cm^−1^, with decreased intensity. These results indicated that the amino groups and carboxylate groups of peptides might be involved in the covalent binding reaction with calcium ions (33). Furthermore, the wave numbers at 1,450.13, 1,203.22, and 1,071.28 cm^−1^ shifted to 1,447.44, 1,319.69, and 1,101.95 cm^−1^, respectively, when bound with calcium, it might be attributed to the carbonyl oxygen contribute to the calcium chelate activity. Similar observations in the spectra were found in schizochytrium protein hydrolysates bound with calcium (16). The absorption peak of 3,346.21 cm^−1^ moved to the higher frequency of 3,384.49 cm^−1^ in the chelation procedure, indicating that hydrogen bonds were replaced with N–Ca bonds (34). In addition, the absorption band at 995.09 cm^−1^ corresponding to P-O-C stretching vibrations reduced to 990.22 cm^−1^ after chelation, which was attributed to the combination of P–O with calcium (35).

The results confirmed that the carboxyl oxygen group, amino group, and phosphoric acid group might be involved in the chelate reaction of SPP and calcium ions.

#### 3.3.3. Conformation analysis

Circular dichroism (CD) spectra are a classical method for fast determination of peptide secondary structure. Figure 3C shows that the presence of calcium induces different conformations. One negative peak emerged at ~196 nm in the original peptide, which demonstrated random coil conformation (36). After chelating, the secondary structure of SPP changed as follows: the α-helix, β-turn, and random coil conformation contents decreased, whereas the percentage of β-sheet increased.

### 3.4. Thermal stability analysis of SPP-Ca

**Figure 4:**
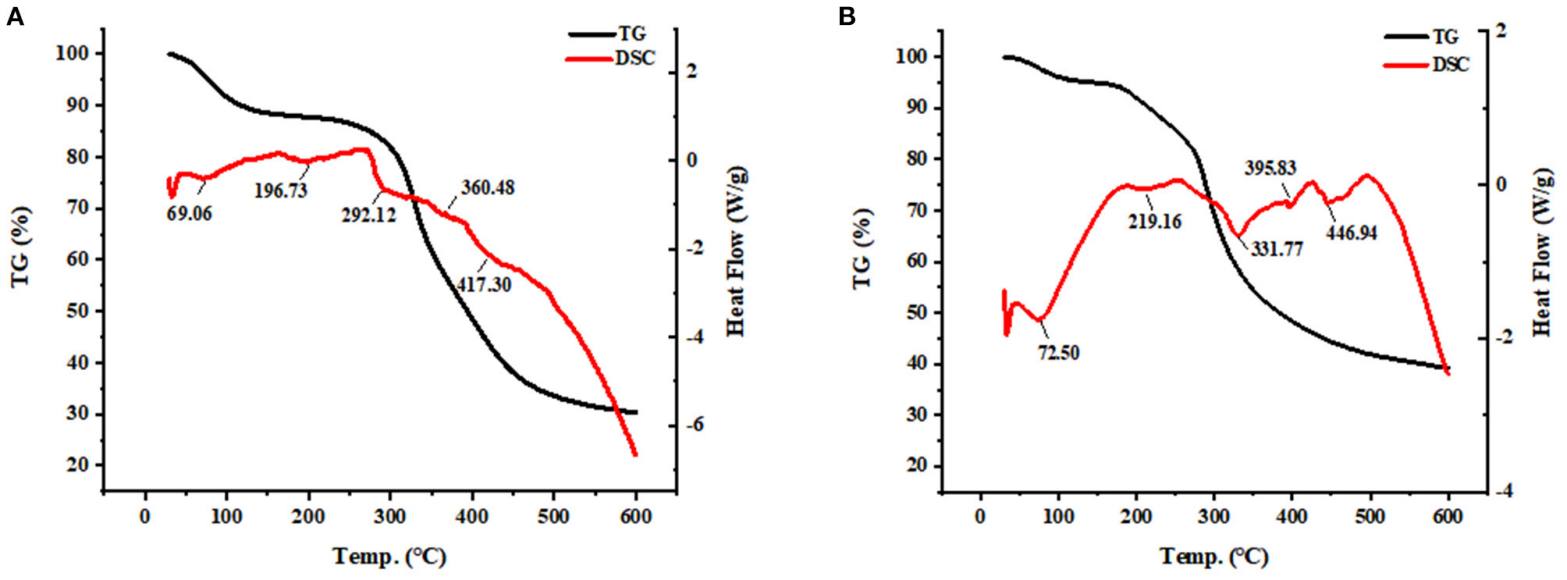
TG-DSC analysis of SPP **(A)** and SPP-Ca **(B)**.

The thermostability changes between SPP and SPP-Ca chelate are presented in Figure 4. The thermal decomposition reaction of SPP underwent 76.35% weight loss throughout the process, and the Td value of SPP corresponding to the three stages was 69.05, 196.73, 292.17, 360.48, and 417.30°C, respectively. These existed endothermic peaks were probably due to the C–N bond groups in SPP (37). Whereas, for SPP-Ca, some prominent shift endothermic peaks were observed at 72.50, 219.16, 331.77, 395.83, and 446.94°C accompanied by greater weight loss.

### 3.5. Effect of SPP and SPP-Ca complexes on proliferation and osteogenic differentiation in MC3T3-E1

**Figure 5:**
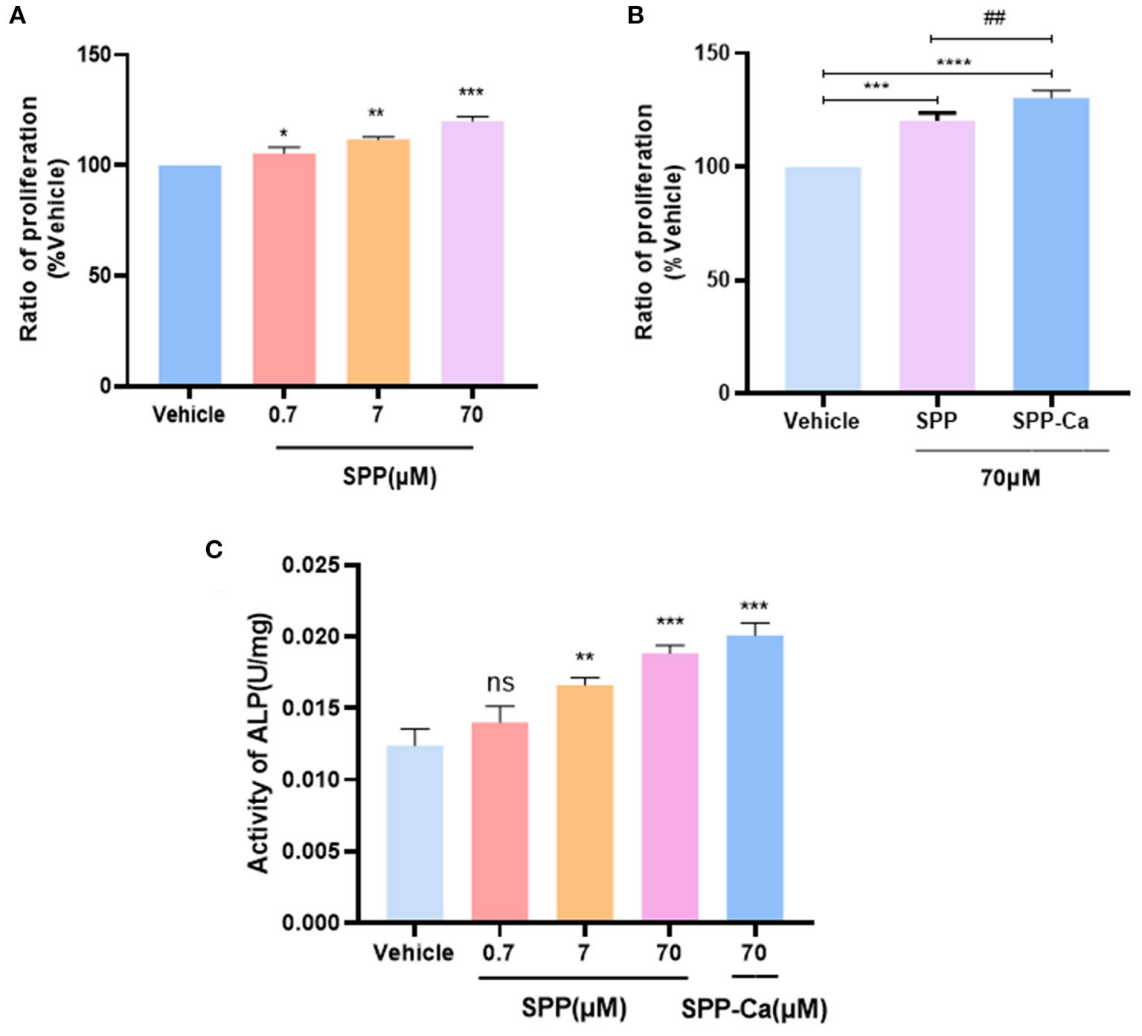
Proliferation of osteoblast response to SPP **(A)** and SPP-Ca **(B)**. MC3T3-E1 was treated with different concentrations (0.7, 7, and 70 μM) of SPP and SPP-Ca (70 μM) for 72h. Changes in the ALP activity of MC3T3-E1 treated with different concentrations of SPP and SPP-Ca **(C)**. *n* = 5. Data are presented as means ± SEMs and analyzed by one-way ANOVA followed by Tukey's multiple comparison test. *p < 0.05, **p < 0.01, and ***p < 0.001 vs. vehicle. ^*##*^Indicated significant differences, *p* < 0.05.

As shown in Figure 5A, the MTT assay indicated that SPP dose-dependently increase the cell viability of MC3T3-E1 cells. The greatest facilitating proliferation osteoblast activity was observed in 70 μM SPP reaching 143.52% with no obvious cytotoxic effect. Interestingly, SPP-Ca achieved stronger activity to promote the proliferation osteoblast at an equal concentration (Figure 5B). Then, we further investigated the biological effects of SPP-Ca on differentiation. The ALP activity reached 0.014, 0.015, 0.018, and 0.020 U/mg at 0.7, 7, and 70 μM SPP treatment, respectively (Figure 5C). In summary, 70 μM SPP significantly increased the osteogenic differentiation of MC3T3-E1. Therefore, the SPP-Ca group used a fixed concentration in the further experiment. Treatment with 70 μM SPP-Ca resulted in a significant increase in the ALP activity of MC3T3-E1 cells when compared with the control group.

## 4. Discussion

The motivation for this study stemmed from a previous study on soy peptide (DEDEQIPSHPPR) stimulating osteoblast differentiation (22). Interestingly, current published literature highlighted the crucial role of phosphorylated modification in calcium binding or further absorption, offering theoretical support for the efficacy and feasibility of the modification that we performed. In this study, phosphorylated soy peptide SPP (DEDEQIPPHPPR) was innovatively synthesized and employed as materials, and the possible interaction mechanism between the SPP and calcium, stability of SPP-calcium complexes, and their promoting osteogenesis activity were comprehensively investigated.

The chemically synthesized SPP with a sequence of Asp-Glu-Asp-Glu-Gln-Ile-Pro-Ser-O-${\text{PO}}_{3}^{2-}$-His-Pro-Pro-Arg (DEDEQIPPHPPR, 1,499.43) had a calcium-binding activity of 50.24 ± 0.20 mg/g. Generally, it is well established that the binding ability of calcium ions for peptides not only depends on the length and net charge of peptides but also on specific amino acid groups and amino acid composition (16, 38). The functional calcium-binding site in peptide bond contains an O atom of the carbonyl group and an N atom of the amino group or imino group. Liu et al. (26) suggested that wheat germ protein-derived calcium-binding peptides are rich in Glu, Asp, and Arg. Bao et al. (27) pointed out that the calcium-binding site of polypeptide might be carboxyl groups of Asp and Glu. Consequently, both the Asp and Glu residues in the SPP might contribute to chelation with calcium ions. Though phosphorylation of serine residues might provide suitable binding sites for positively charged metals. However, previous studies have shown that phosvitin phosphopeptides (39), herring egg phosphopeptides (40), and CPPs (41) were 468 ± 152.80, 90.08 ± 2.02, and 60.17 mg/g, respectively, much higher than that of the SPP isolated from soy yogurt. When calcium content reached a high level, the steric hindrance of phosphate groups might be strengthened, which could not be conducive to the chelation of SPP with amino groups and carboxyl groups, so similar calcium-binding ability cannot be achieved.

Molecule docking results showed that the SPP possesses certain calcium-binding abilities (Figure 1). Generally speaking, the combination between macromolecules and small ligands is driven by non-covalent bonds involving hydrogen bonding, van der Waals forces, hydrophobic interactions, and electrostatic attractions, which are often determined by thermodynamic parameter values (42). Through ITC measurement, it can be concluded that chelation produced an exothermic reaction of 4.224 kJ/mol. In addition, the exothermic reaction yielded by the formation of a coordination bond between Ca^2+^ and phosvitin exerted a negative effect on ΔH. Similar results were also observed in the binding of calcium to VHS(p)S(p) and VLPVPQK (43, 44). When ΔH < 0 and Δ s > 0, the electrostatic forces dominated. Moreover, the estimated stoichiometric ratio of SPP and calcium was 1:1, which also occurred in EDLALLEK peptide that was reported by Sun where the binding of EDLALLEK peptide to Ca^2+^ occurred mostly through the carboxyl oxygen atom of Glu and Asp at a ratio of 1:1 (17).

To clarify the interaction patterns between SPP and calcium, a variety of spectral analyses have been used. Both UV and FTIR spectra results indicated that there was a complex of SPP and calcium via the carboxyl oxygen group, amino group, and phosphoric acid group. Corresponding conformational changes are shown in Figure 3C. The increase in β-turn under the presence of Ca^2+^ greatly docks of peptide in the hydrophobic cavity and ultimately promote the transport of Ca-chelated form across the plasma membrane, indicating that the chelation reaction between SPP and calcium produced a much more ordered and compact structure (45, 46). Considering previous research, chelation does change the structure of SPP, and it will affect thermal stability. Thus, additional study is required to confirm this possibility.

Compared with SPP, the chemical bonds breaking of SPP-Ca required higher energy-generating alterations in thermal denaturation temperature. Chelation enhanced the thermal stability of SPP which gives unique advantages in the applications in the functional food field. Many studies also have reported that the application of naturally bioactive peptide calcium chelates generally positively regulating the proliferation and differentiation of osteoblasts, thus preventing bone loss (47, 48). Cell proliferation plays a vital role in the first stage of the osteoblast phenotype (49). We also found that SPP-Ca exhibited biological effects on promoting osteogenesis in MC3T3-E1. In our study, SPP-Ca with concentrations ranging from 0.7 to 70 μM was added for 72 h. MTT assay indicated that SPP-Ca showed a concentration-related stimulation in response to increase cell viability. Similar results were also obtained for the binding of calcium to bovine collagen peptides and chum salmon skin gelatin hydrolysates (50, 51). Moreover, ALP activity was the most widely recognized biochemical marker for osteoblastic activity (52). It was worth noting that the ALP activity of peptide increased slightly in the presence of calcium. Taken together, SPP-Ca treatment positively regulated osteoblast proliferation and differentiation.

## 5. Conclusion

In conclusion, a specific soy yogurt peptide with calcium-chelating ability was chemically synthesized with phosphorylation modification, and its binding mechanism was investigated. The results showed that SPP chelated with calcium mainly through carboxyl oxygen of Glu, Asp, and Arg at a stoichiometric ratio of 1:1 and phosphoric acid group, resulting in the formation of a stable complex. SPP-Ca chelate exhibited superior effects in promoting osteoblast proliferation and differentiation than peptide alone *in vitro* assays. Additionally, the peptide can be simply and directly synthesized, which is conducive to future large-scale production of SPP-Ca as a calcium supplement. However, further research is also required to identify the therapeutic effects of SPP-Ca against bone diseases.

## Data availability statement

The original contributions presented in the study are included in the article/Supplementary material, further inquiries can be directed to the corresponding authors.

## Author contributions

XK and ZX were responsible for conceptualizing and designing this study, validation, data curation, and writing—original draft preparation. YC, MD, and ZZ played an important role in software and formal analysis. BX and ZW participated in investigation and visualization. YC, TY, and JG contributed to resources, writing—review and editing, supervision, project administration, and funding acquisition. All authors have read and agreed to the published version of the manuscript.

## Supplementary material

Supplementary Figure 1 Identifications of SPP [DEDEQIPS(P)HPPR] by analytical reversed-phase HPLC **(A)** and HPLC-MS **(B)**.
