# Supplementary figures and images for: Calcium-binding properties, stability, and osteogenic ability of phosphorylated soy peptide-calcium chelate
Source: Front Nutr. 2023 Apr 21;10:1129548. doi: 10.3389/fnut.2023.1129548 (PMC10160607; doi:10.3389/fnut.2023.1129548)

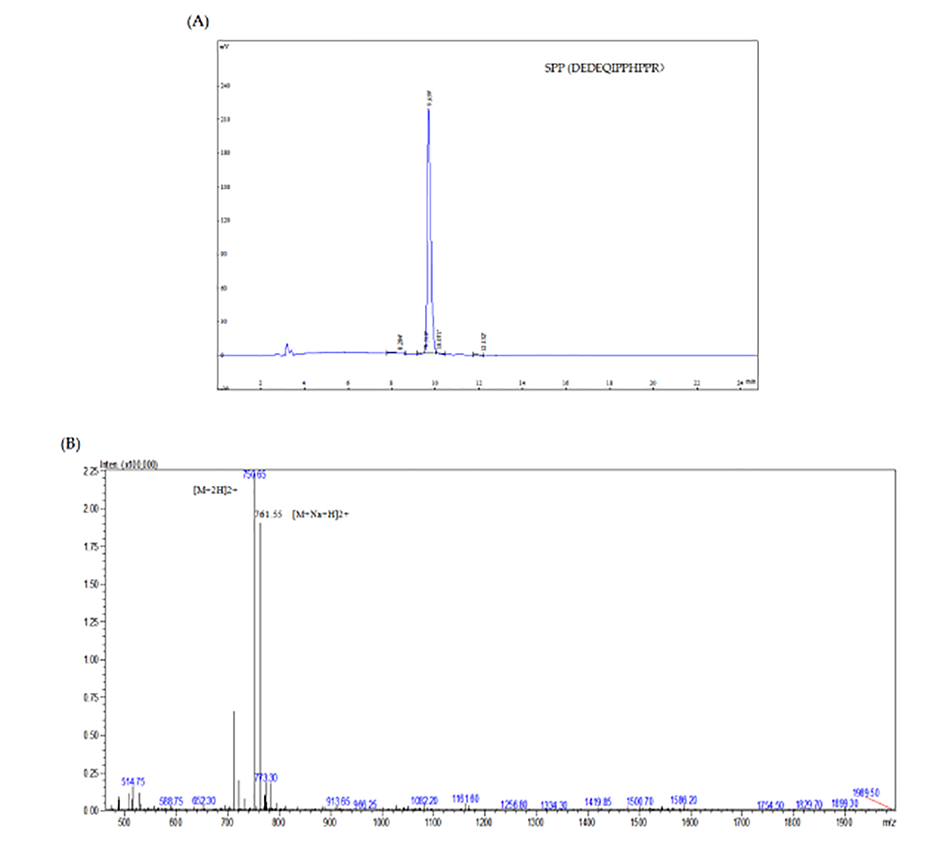

Supplement: Supplementary Figure 1 — Identifications of SPP [DEDEQIPS(P)HPPR] by analytical reversed-phase HPLC (A) and HPLC-MS (B). [file Image_1.TIF]
